# The accuracy and intra- and interobserver variability of PSMA PET/CT for the local staging of primary prostate cancer

**DOI:** 10.1007/s00259-024-06594-0

**Published:** 2024-01-26

**Authors:** Maarten L. Donswijk, Rosemarijn H. Ettema, Dennie Meijer, Maurits Wondergem, Zing Cheung, Elise M. Bekers, Pim J. van Leeuwen, Roderick C. N. van den Bergh, Henk G. van der Poel, André N. Vis, Daniela E. Oprea-Lager

**Affiliations:** 1 Department of Nuclear Medicine, The Netherlands Cancer Institute - Antoni van Leeuwenhoek, Amsterdam, The Netherlands; 2 Department of Urology, Amsterdam University Medical Centres, Vrije Universiteit Amsterdam, Amsterdam, The Netherlands; 3 Department of Radiology and Nuclear Medicine, Amsterdam University Medical Centres, Vrije Universiteit Amsterdam, Amsterdam, The Netherlands; 4 Prostate cancer network, Amsterdam, The Netherlands; 5 Department of Urology, St. Antonius Hospital, Nieuwegein, The Netherlands; 6 Department of Radiology and Nuclear Medicine, University Medical Centre Utrecht, Utrecht, The Netherlands; 7 Department of Pathology, The Netherlands Cancer Institute - Antoni van Leeuwenhoek, Amsterdam, The Netherlands; 8 Department of Urology, The Netherlands Cancer Institute - Antoni van Leeuwenhoek, Amsterdam, The Netherlands

**Keywords:** Prostatic Neoplasms, Neoplasm Staging, Positron Emission Tomography Computed Tomography, Observer Variation

## Abstract

**Purpose:**

Prostate-specific membrane antigen (PSMA) positron emission tomography/ computed tomography (PET/CT) is recognized as the most accurate imaging modality for detection of metastatic high-risk prostate cancer (PCa). Its role in the local staging of disease is yet unclear. We assessed the intra- and interobserver variability, as well as the diagnostic accuracy of the PSMA PET/CT based molecular imaging local tumour stage (miT-stage) for the local tumour stage assessment in a large, multicentre cohort of patients with intermediate and high-risk primary PCa, with the radical prostatectomy specimen (pT-stage) serving as the reference standard.

**Methods:**

A total of 600 patients who underwent staging PSMA PET/CT before robot-assisted radical prostatectomy was studied. In 579 PSMA positive primary prostate tumours a comparison was made between miT-stage as assessed by four nuclear physicians and the pT-stage according to ISUP protocol. Sensitivity, specificity and diagnostic accuracy were determined. In a representative subset of 100 patients, the intra-and interobserver variability were assessed using Kappa-estimates.

**Results:**

The sensitivity and specificity of the PSMA PET/CT based miT-stage were 58% and 59% for pT3a-stage, 30% and 97% for ≥ pT3b-stage, and 68% and 61% for overall ≥ pT3-stage, respectively. No statistically significant differences in diagnostic accuracy were found between tracers.

We found a substantial intra-observer agreement for PSMA PET/CT assessment of ≥ T3-stage (k 0.70) and ≥ T3b-stage (k 0.75), whereas the interobserver agreement for the assessment of ≥ T3-stage (k 0.47) and ≥ T3b-stage (k 0.41) were moderate.

**Conclusion:**

In a large, multicentre study evaluating 600 patients with newly diagnosed intermediate and high-risk PCa, we showed that PSMA PET/CT may have a value in local tumour staging when pathological tumour stage in the radical prostatectomy specimen was used as the reference standard. The intra-observer and interobserver variability of assessment of tumour extent on PSMA PET/CT was moderate to substantial.

**Supplementary Information:**

The online version contains supplementary material available at 10.1007/s00259-024-06594-0.

## Introduction

Prostate cancer (PCa) is the most common cancer in men of middle and older age [1]. Adequate local staging (T-stage) in primary PCa is important to guide treatment decisions. Such as surgical planning (including nerve-sparing options) in patients undergoing radical prostatectomy or in those who opt for external radiotherapy of the prostate. Curation is more likely achieved when disease is limited to the prostate gland (i.e., T-stage ≤ 2).

The presence of T3-disease (i.e. T3a in case of extracapsular extension, or T3b in case of seminal vesicle invasion [2]) may be assessed by a combination of serum prostate specific antigen (PSA), digital rectal examination, transrectal ultrasound, the outcome of prostate biopsies and by magnetic resonance imaging (MRI). Although MRI is the most accurate single staging modality with high specificity (82–88%), it’s overall accuracy to detect T3-disease is hampered by its lower sensitivity (51–57%) [3, 4]. Therefore, efforts may be made to improve accuracy of detecting T3-disease.

Prostate-specific membrane antigen (PSMA) positron emission tomography / computed tomography (PET/CT) is increasingly being used for initial staging of patients with intermediate and high-risk primary PCa as well as in patients with recurrent disease after treatment with curative intent [5–7]. While PSMA-PET is an adequate modality for staging metastases, it may also be applied for the assessment of the local extent of primary PCa. Several studies on the assessment of local staging on PSMA PET showed promising results [8]. However, these studies were performed in single centre settings and had a limited number of patients, thus possibly influencing the statistical robustness. Also, in these studies almost exclusively ^68^Ga-labelled PSMA tracers were used, which may question its generalizability to the ^18^F-labelled PSMA tracers.

Therefore, we assessed a large, multicentre cohort of patients with intermediate and high-risk PCa using both ^68^Ga- and ^18^F-labelled PSMA tracers. The diagnostic accuracy of molecular imaging local tumour staging by PSMA PET/CT (miT-stage) was determined with the radical prostatectomy specimen (pT-stage) serving as the reference standard. Furthermore, as an important determinant of diagnostic test reliability, both intra-observer and interobserver variability of PSMA PET/CT for local tumour staging were assessed.

## Material and methods

This retrospective cohort study was conducted by the Amsterdam UMC and the Netherlands Cancer Institute – Antoni van Leeuwenhoek (NCI-AVL) as tertiary referral centres within the Prostate Cancer Network the Netherlands. Approval of the institutional review board of both hospitals was obtained for this study, meanwhile waiving the need to receive informed consent (VUmc2019.586 and IRBd20-041).

### Patients

A cohort of 600 consecutive patients with intermediate and high-risk primary PCa was studied. All patients underwent PSMA PET/CT before robot-assisted radical prostatectomy (RARP) and extended pelvic lymph node dissection (ePLND) between August 2016 and June 2021. From all patients, clinical and pathological data were obtained from a prospectively maintained comprehensive database. Patients were classified according to the D’Amico risk classification into having low, intermediate, or high-risk disease [9]. Patients underwent a PSMA PET/CT as per protocol because of the presence of intermediate and high-risk factors (i.e., ≥ clinical T3, an International Society of Urologic Pathologists (ISUP) grade 3 / Gleason score ≥ 4 + 3 = 7, and/or a serum PSA ≥ 20 ng/mL).

### Pre-operative PSMA PET imaging

PSMA PET imaging was performed in NCI-AVL, Amsterdam UMC or in one of the referring external centres using multiple tracers, according to local protocols on EARL accredited PET systems [10, 11] and in line with EANM guidelines on prostate cancer imaging [12, 13]. The applied PSMA tracers included the ^18^F-labelled tracers: [^18^F]DCFPyL, [^18^F]PSMA-1007, [^18^F]-JK-PSMA-7, and the ^68^Ga-labelled tracer [^68^Ga]Ga-PSMA-11. The ^18^F-labelled tracers were synthesized via direct radiofluoration at an on-site cyclotron facility. [^68^Ga]Ga-PSMA-11 was produced on‐site using a fully automated system (Scintomics GmbH), compliant to the Good Manufacturing Practices-guidelines. PET-images were acquired from mid-thigh to skull-base using Siemens Truepoint, Philips Ingenuity / Gemini TF, or Philips Vereos integrated PET/CT systems. PET-images were combined with either a low-dose CT-scan (120–140 kV, 40–80 mAs with dose modulation) or a full dose CT-scan (130 kV, 110mAs), with or without intravenous contrast enhancement. All PET-images were corrected for scatter, decay, and random coincidences.

### Image analysis and molecular image tumour-stage assessment

**Fig. 1:**
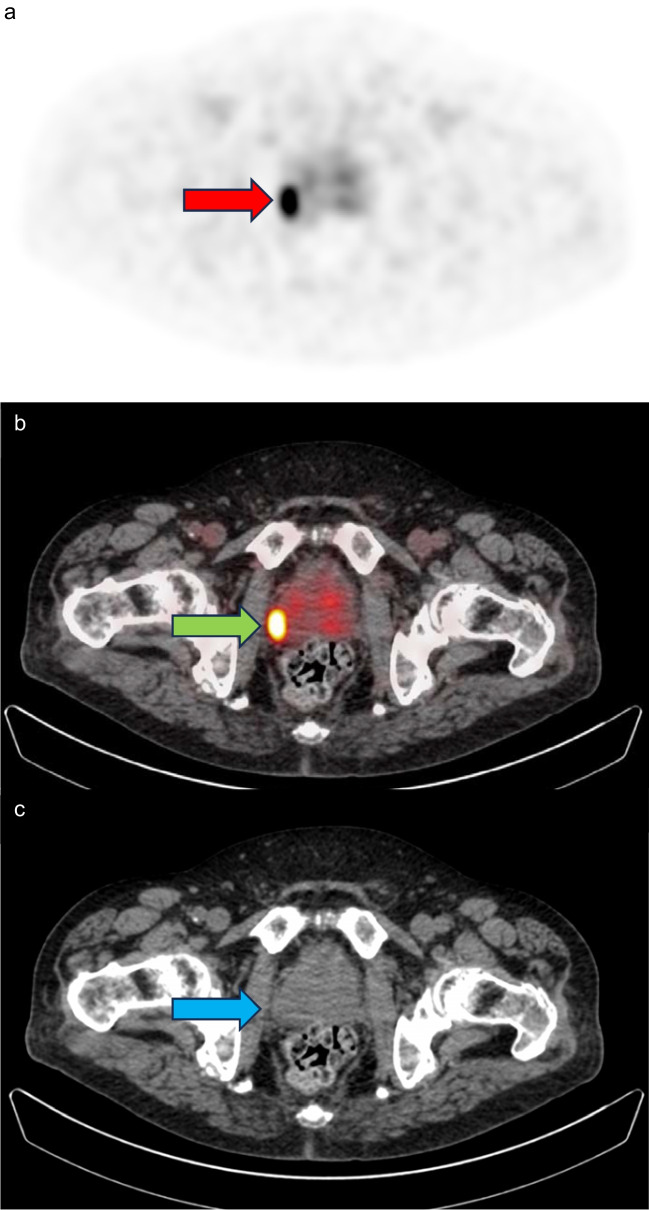
Example of PSMA PET based assessment of T3a-stage. A 78-year-old man presented with a clinical T1c ISUP grade 2 prostate cancer and an iPSA of 7.0, who underwent PSMA PET/ non-contrast enhanced CT using [^18^F]-JK-PSMA-7 as tracer because of a suspicious pelvic node on MRI. Attenuation-corrected PET reconstruction (**a**), fused PET – non-contrast enhanced CT (**b**) and non-contrast enhanced CT (**c**) at the same axial level of the prostate. These show an intensely PSMA-positive prostate tumour in the peripheral zone on the right (red arrow) and PET tumour activity extending outside the prostate contour as depicted on CT (green arrow), as well as asymmetrical bulging of the prostate contour on CT (blue arrow). According to the standardized assessment, a molecular imaging tumour-stage 3a (Likert score 2) was assigned. The patient underwent a robot-assisted radical prostatectomy after which a histopathologically confirmed pT3a, ISUP grade 3 acinar adenocarcinoma of the prostate with cribriform growth was found

**Fig. 2:**
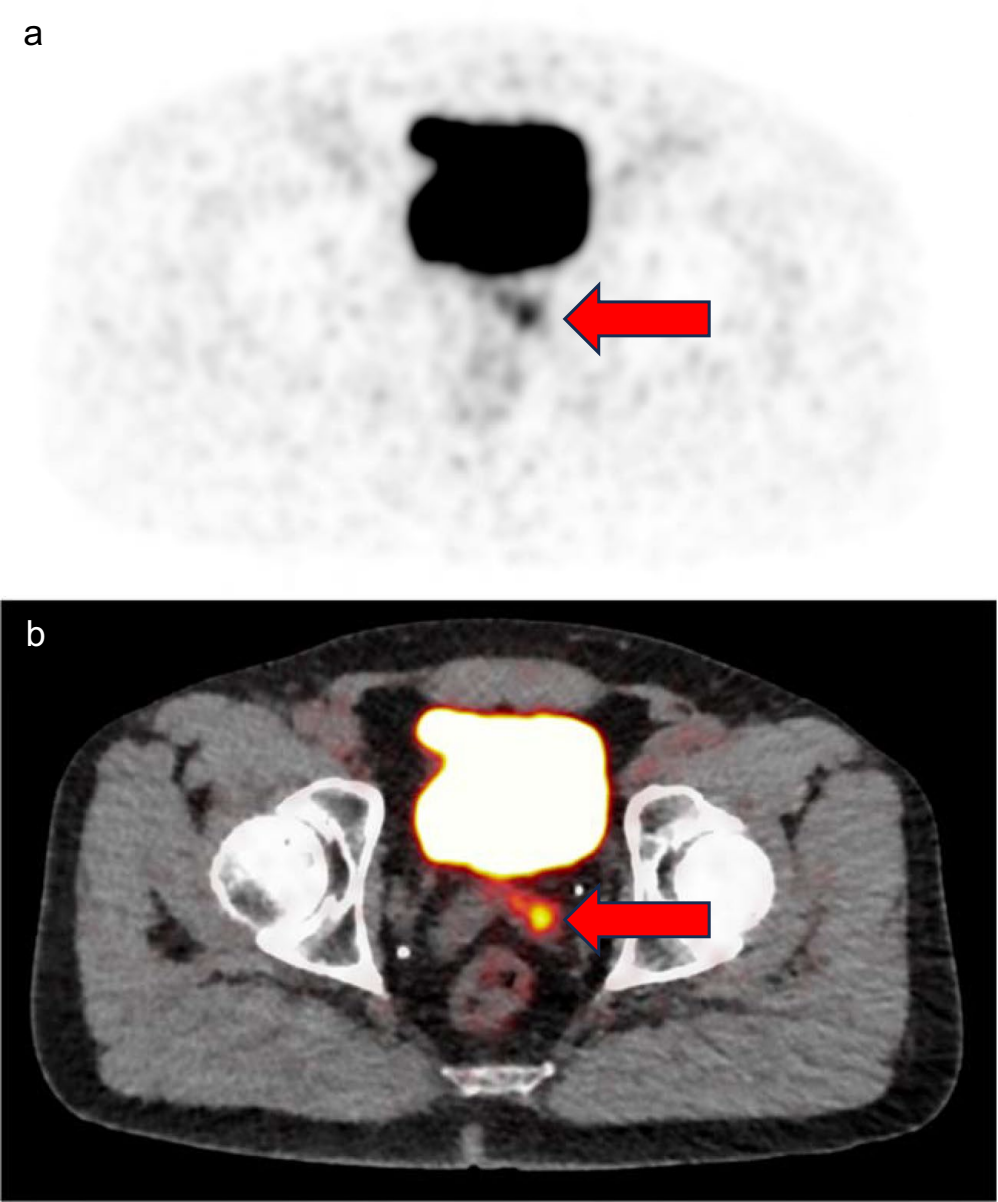
Example of PSMA PET based assessment of T3b-stage. A 61-year-old man presented with a clinical T2a ISUP grade 2 prostate cancer and an iPSA of 23.0, who underwent PSMA PET/ non-contrast enhanced CT using [^68^Ga]Ga-PSMA-11 as tracer because of the presence of a high-risk factor (iPSA > 20 ng/ml). Attenuation-corrected PET reconstruction (**a**) and fused PET – non-contrast enhanced CT (**b**) at the same axial level of the prostate show an area of elevated uptake in the left seminal vesicle (red arrows). According to the standardized assessment, a molecular imaging tumour-stage 3b was assigned. The patient underwent a robot-assisted radical prostatectomy after which a histopathologically confirmed pT3b, ISUP grade 2 acinar adenocarcinoma of the prostate with cribriform growth was found

Current guidelines advise to report a molecular imaging (mi) based T-stage which is a PSMA-PET based derivation of the clinicopathological TNM system [14–16]. In this visual image assessment system, the following miT-stages are distinguished: miT0 (no tumour visible in the prostate), miT2 (tumour localized within the prostate capsule), miT3a (extracapsular extension (ECE)), miT3b (seminal vesicle invasion (SVI)), miT4 (extension in other extraprostatic tissues such as urinary bladder, rectum, or other pelvic structures). However, in these guidelines, clear criteria specifically for the assessment of miT3a- and miT3b-stage are yet lacking. Therefore, four observers (nuclear medicine physicians with ample experience in PSMA PET/CT reading, i.e., more than 5 years of experience in PSMA PET/CT reading according to E-PSMA guidelines [14] and more than 2000 assessed PSMA PET/CT scans: DO (Amsterdam UMC), and MD, MW, ZC (NCI-AVL)), reached consensus on further specified criteria for miT3a- and miT3b-stage, based on earlier reported criteria and personal experience [17]. According to this consensus, miT3a was determined as either PET tumour activity extending beyond the borders of the prostate contour as visualized on concurrent CT and/or the presence of morphological criteria on CT, e.g., an angulated contour of the prostate gland or obliteration of the rectoprostatic angle (Fig. 1), and miT3b was determined as PET tumour activity extending beyond the prostate base into the seminal vesicle and /or separate focal activity in the seminal vesicle (Fig. 2).

Next, all PSMA PET/CT scans were analysed by the four nuclear medicine physicians (150 cases each) for PSMA expression in the local prostate tumour and a PSMA-expression score was assigned according to E-PSMA guidelines [14]. According to the consensus-based criteria, a miT-stage was assigned distinguishing the following stages: miT0, miT2, miT3a, miT3b or miT4. Regarding the presence of extra-capsular extension (miT3a), readers designated the presence using a 3-point Likert scale as 0. not likely (miT2-stage); 1. possible (possible miT3a); and 2. probable (miT3a). Scans in which no PSMA positive prostate tumour was observed (PSMA expression score 0 / miT0 stage) were deemed not suitable for local staging and were left out of the further comparative analyses to pathological tumour stage. Observers were blinded from all clinical information, including the clinical T-stage, clinical reports of the scans, and results of other imaging.

### Analysis of intra- and interobserver variability of molecular image tumour-stage assessment

To determine the intra-observer variability of miT-stage assessment, each observer re-assessed a random selection of 25 previously evaluated cases (i.e., 100 in total) at least 6 months after the initial assessments. In this, the observers were blinded to previous individual assessments.

The interobserver variability of miT-stage assessment was determined by dual observer analysis of 25 randomly selected cases per observer (i.e., 100 in total) using varying pairs of observers. The observers were blinded to earlier assessments.

### Histopathological assessment of pathological T-stage after RARP

Histopathology was considered the reference standard for tumour stage (i.e., pT-stage) of the primary PCa. All RARP specimens were processed and reported according to the ISUP standard protocols by specialized uropathologists [18] and a pathological T-stage was assigned according to TNM (8^th^ ed.) [2].

### Statistical analysis

Statistical analyses were performed using the Statistical Package for Social Sciences (SPSS, IBM; v26). Continuous variables are expressed as mean (± standard deviation (SD)), or in case of not normally distributed data as median (interquartile range (IQR)). Categorical variables are presented with absolute and relative frequencies.

The diagnostic accuracy of preoperative PSMA PET/CT-based molecular imaging tumour stage for predicting pathological tumour stage (pT-stage) as assessed within the radical prostatectomy specimen was determined. In this, sensitivity, specificity, negative predictive value and the positive predictive value were assessed. Furthermore, as a measure of diagnostic accuracy, areas under the receiver operating characteristics curves (AUC) were calculated.

The diagnostic accuracy was determined for all observers combined (observer-average) as well as for individual observers (observer-specific). Furthermore, the diagnostic accuracy was determined per tracer (tracer-specific). For calculation of diagnostic accuracy, data were dichotomized treating Likert scale points 0 as negative, and 1 and 2 as positive for miT3a; stages miT3b and miT4 were grouped; and overall ≥ miT3 was determined as any miT3a, miT3b or miT4. Pathological tumour stage was assessed as pT2, pT3a, and ≥ pT3b. Overall ≥ pT3 was determined as any pT3a or ≥ pT3b.

Intra- and interobserver agreement of PSMA PET/CT assessment on the presence of ≥ T3-stage were estimated using Cohen’s weighted kappa with 95% CI, discerning categories T2 (Likert 0), possible ≥ T3 stage (Likert 1) and probable ≥ T3 stage (Likert 2). Intra- and interobserver agreement on ≥ T3b-stage were estimated separately using Cohen’s kappa with 95% CI.

As conventionally classified, kappa values of 0–0.20 indicate a poor, 0.21–0.40 a fair, 0.41–0.60 a moderate, 0.61–0.80 a substantial, and 0.81–1.0 a nearly perfect agreement [19]. All statistical tests were two-tailed, and a value of *p* ≤ 0.05 was considered statistically significant.

## Results

### Patient characteristics

The 600 included patients who underwent RARP and preoperative PSMA PET/CT had a median (IQR) age of 68 (64–72) years and a median (IQR) initial PSA of 11 ng/ml (7–20). Of these, 371 (61.8%) had a T3-stage at final pathology. Further clinical and pathological characteristics are listed in Table 1.

**Table 1:** Clinical and histopathological characteristics of 600 evaluated patients who underwent PSMA PET/CT imaging before robot-assisted radical prostatectomy

	*n* = 600
Age (years), median (IQR)	68 (64–72)
Initial PSA level (ng/ml), median (IQR)	11 (7–20)
Time between PSMA PET/CT and RARP (days), median (IQR)	58 (37–80)
D’Amico Risk Classification;	
Low Risk	2 (0.3%)
Intermediate Risk	162 (27.0%)
High Risk	436 (72.7%)
Pathological Grade Group in prostatectomy specimen;	
ISUP 1	7 (1.2%)
ISUP 2	189 (31.5%)
ISUP 3	203 (33.8%)
ISUP 4	57 (9.5%)
ISUP 5	144 (24.0%)
Pathological tumour stage	
pT2	229 (38.2%)
pT3 (overall)	371 (61.8%)
• *pT3a*	• *227 (37.8%)*
• ≥ *pT3b*	• *144 (24.0%)*

*SD* = standard deviation; *IQR* = interquartile range; *PSA* = prostate-specific antigen; *PSMA* = prostate-specific membrane antigen; *PET* = positron emission tomography; *CT* = computed tomography; *RARP* = robot-assisted laparoscopic radical prostatectomy; *ISUP* = International Society of Urological Pathology; *p* = pathology; *T* = tumour-stage

### PSMA PET/CT staging characteristics

Patients were scanned using four different PSMA tracers, namely [^68^Ga]Ga-PSMA-11 in 287 (47.8%), [^18^F]DCFPyL in 210 (35.0%), [^18^F]PSMA-1007 in 70 (11.7%), and [^18^F]-JK-PMSA-7 in 33 (5.5%) out of 600 patients. The doses and biodistribution times of the different tracers are provided in Table 2. Molecular Imaging primary tumour staging (miT-stage) on PSMA PET/CT is listed in Table 3. In 21 (3.5%) out of 600 patients, no PSMA positive primary prostate tumour was observed (PSMA expression score 0 / miT0). These cases were left out of the analyses on the diagnostic accuracy of PSMA PET/CT for pT-staging. In the remaining 579 patients, miT2 was assessed in 250 (41.7%), miT3a-stage was observed in 276 (46.0%), while miT3b/miT4 stage was found in 53 (8.8%) patients.

**Table 2:** PSMA PET/CT imaging characteristics in 600 evaluated patients

PSMA PET/CT tracer	*n* (%)	Tracer dose (MBq, median (IQR))	Tracer biodistribution time (minutes, median (IQR))
[^68^Ga]Ga-PSMA-11	287 (47.8%)	109 (97–149)	50 (45–60)
[^18^F]DCFPyL	210 (35.0%)	217 (201–303)	73 (58–118)
[^18^F]PSMA-1007	70 (11.7%)	284 (259–312)	84 (75–91)
[^18^F]-JK-PSMA-7	33 (5.5%)	292 (255–324)	90 (83–90)
PSMA PET combined with			
low-dose, non-ceCT	408 (68.0%)		
high-dose, ceCT	134 (22.3%)		
high-dose, non-ceCT	58 (9.7%)		

*PSMA* = prostate-specific membrane antigen; *PET* = positron emission tomography; *CT* = computed tomography; *ceCT* = contrast-enhanced CT; *MBq* = megabecquerel; *IQR* = interquartile range

**Table 3:** PSMA PET/CT primary tumour staging characteristics in 600 evaluated patients

PSMA expression score of prostate tumour	
1	187 (31.2%)
2	244 (40.7%)
3	148 (24.7%)
miT-stage	
miT2	250 (41.7%)
miT3a	276 6.0%)
• *Likert 1*	• *128 (21.3%)*
• *Likert 2*	• *148 (7.2%)*
miT3b—miT4	53 (8.8%)
PSMA expression score 0 / miT0	21 (3.5%)

*PSMA* = prostate-specific membrane antigen; *PET* = positron emission tomography; *CT* = computed tomography; *mi* = molecular imaging; *T* = tumour-stage

### Diagnostic accuracy of PSMA PET for pathological T-staging

In the 579 patients with a PSMA positive primary tumour, the observer-average sensitivity and specificity of PSMA PET/CT-based miT-stage for predicting pT3a-stage, as assessed in the radical prostatectomy specimen, were 58% and 59%, respectively. For ≥ pT3b-stage these were 30% and 97%, and 68% and 61% for overall ≥ pT3-stage, respectively. The PPV and NPV of miT-stage were 46% and 70% for pT3a-stage, 77% and 81% for ≥ pT3b-stage, and 73% and 54% for overall ≥ pT3-stage, respectively (Table 4). Diagnostic accuracy expressed by means of AUC showed an AUC of 0.59 (95% CI 0.54—0.63) for predicting pT3a-stage, 0.64 (95% CI 0.58—0.69) for predicting pT3b-stage, and 0.64 (95% CI 0.60—0.69) for predicting overall ≥ pT3-stage.

**Table 4:** Observer-average diagnostic accuracy of PSMA PET/CT based tumour stage (miT-stage) for pathological tumour stage (pT-stage) in 579 patients with a PSMA-positive prostate tumour undergoing pre-operative PSMA PET/CT imaging and concurrent robot assisted radical prostatectomy (RARP) as reference standard, regarding **A**. pT3-stage; **B**. ≥ pT3b-stage and **C**. ≥ pT3-stage

A			
	no pT3a; *n*	pT3a; *n*	Total; (PPV/NPV)
no miT3a	212	91	303 (70% NPV, 95% CI 65–75%)
miT3a	148	128	276 (46% PPV, 95% CI 41–52%)
Total	360 (59% specificity, 95% CI 54–64%)	219 (58% sensitivity, 95% CI 52–65%)	579
	AUC 0.59 [95% CI 0.54—0.63]		
B			
	< pT3b; *n*	≥ pT3b; *n*	Total; (PPV/NPV)
< miT3b	429	97	526 (82% NPV, 95% CI 78–85%)
miT3b – miT4	12	41	53 (77% PPV, 95% CI 65–87%)
Total	441 (97% specificity, 95% CI 96–99%)	138 (30% sensitivity, 95% CI 23–38%)	579
	AUC 0.64 [95% CI 0.58—0.69]		
C			
	pT2; *n*	≥ pT3; *n*	Total; (PPV/NPV)
miT2	135	115	250 (54% NPV, 95% CI 48–60%)
≥ miT3	88	241	329 (73% PPV, 95% CI 68–78%)
Total	223 (61% specificity, 95% CI 54–67%)	356 (68% sensitivity, 95% CI 63–72%)	579
	AUC 0.64 [95% CI 0.60—0.69]		

*p* = pathology; *mi* = molecular imaging; *T* = tumour-stage; *PPV* = positive predictive value; *NPV* = negative predictive value; *AUC* = area under the receiver operator curve; *CI* = confidence interval

The observer-specific diagnostic accuracy of PSMA PET/CT-based miT-stage ranged from 0.54 to 0.65 for predicting pT3a-stage, from 0.58 to 0.78 for predicting ≥ pT3b-stage, and from 0.59 to 0.75 for predicting overall ≥ pT3-stage. Further details are presented in Table 5.

**Table 5:** Observer-specific diagnostic accuracy of PSMA PET/CT based tumour stage (miT-stage) for pathological tumour stage (pT-stage) in 579 patients with a PSMA-positive prostate undergoing pre-operative PSMA PET/CT imaging and concurrent robot assisted radical prostatectomy (RARP) as reference standard

Parameter / Observer	pT3a	≥ pT3b	≥ pT3
sensitivity (%, [95% CI])			
observer 1	46 [31 – 62]	67 [47–83]	71 [60–82]
observer 2	80 [68 – 89]	31 [18–48]	84 [76–91]
observer 3	53 [41 – 65]	16 [7–28]	59 [49–68]
observer 4	51 [38 – 63]	21 [9–36]	59 [49–68]
specificity (%, [95% CI])			
observer 1	83 [73–90]	89 [82–95]	78 [65–89]
observer 2	38 [28–48]	99 [96–100]	34 [22–48]
observer 3	56 [47–64]	100 [nc]	60 [48–71]
observer 4	65 [55–74]	98 [95–100]	71 [58–82]
PPV (%, [95% CI])			
observer 1	60 [42–76]	64 [45–81]	82 [70–90]
observer 2	47 [37–57]	92 [68–100]	71 [62–78]
observer 3	39 [29–49]	100 [nc]	69 [59–77]
observer 4	49 [37–62]	78 [46–96]	77 [66–86]
NPV (%, [95% CI])			
observer 1	73 [63–82]	91 [83–96]	67 [54–78]
observer 2	73 [59–84]	82 [75–88]	53 [36–70]
observer 3	69 [59–78]	78 [71–84]	49 [39–60]
observer 4	66 [56–76]	80 [73–87]	51 [40–62]
AUC [95% CI]			
observer 1	0.65 [0.53–0.76]	0.78 [0.66–0.90]	0.75 [0.65–0.84]
observer 2	0.59 [0.49–0.68]	0.65 [0.54–0.77]	0.59 [0.49–0.69]
observer 3	0.54 [0.46–0.63]	0.58 [0.48–0.68]	0.59 [0.51–0.68]
observer 4	0.58 [0.48–0.67]	0.59 [0.48–0.71]	0.65 [0.56–0.74]

*p* = pathology; *T* = tumour-stage; *PPV* = positive predictive value; *NPV* = negative predictive value; *AUC* = area under the receiver operator curve; *CI* = confidence interval; *nc* = non-computable

The tracer-specific diagnostic accuracy of PSMA PET/CT-based miT-stage ranged from 0.54 to 0.63 for predicting pT3a-stage, from 0.57 to 0.65 for predicting ≥ pT3b-stage, and from 0.61 to 0.69 for predicting overall ≥ pT3-stage. Further details are presented in Table 6.

**Table 6:** Tracer-specific diagnostic accuracy of PSMA PET/CT based tumour stage (miT-stage) for pathological tumour stage (pT-stage) in 579 patients with a PSMA-positive prostate undergoing pre-operative PSMA PET/CT imaging and concurrent robot assisted radical prostatectomy (RARP) as reference standard

Parameter / Tracer	pT3a	≥ pT3b	≥ pT3
sensitivity (%, [95% CI])			
[^68^Ga]Ga-PSMA-11	67 [57–76]	32 [22–43]	71 [64–77]
[^18^F]DCFPyL	49 [38–60]	30 [17–46]	62 [53–70]
[^18^F]PSMA-1007	67 [52–81]	26 [10–48]	71 [58–83]
[^18^F]-JK-PSMA-7	50 [26–75]	22 [4–54]	68 [48–85]
specificity (%, [95% CI])			
[^68^Ga]Ga-PSMA-11	59 [51–66]	99 [97–100]	59 [49–68]
[^18^F]DCFPyL	59 [50–67]	97 [93–99]	61 [50–71]
[^18^F]PSMA-1007	60 [44–74]	94 [86–99]	65 [45–82]
[^18^F]-JK-PSMA-7	62 [41–80]	92 [78–99]	69 [42–89]
PPV (%, [95% CI])			
[^68^Ga]Ga-PSMA-11	47 [39–55]	92 [77–99]	73 [66–80]
[^18^F]DCFPyL	47 [34–54]	69 [45–88]	70 [60–77]
[^18^F]PSMA-1007	51 [35–67]	63 [29–89]	81 [68–91]
[^18^F]-JK-PSMA-7	47 [24–71]	50 [11–89]	79 [58–93]
NPV (%, [95% CI])			
[^68^Ga]Ga-PSMA-11	77 [69–83]	80 [75–85]	55 [46–64]
[^18^F]DCFPyL	63 [54–72]	86 [80–90]	53 [43–63]
[^18^F]PSMA-1007	60 [42–76]	78 [67–87]	52 [34–69]
[^18^F]-JK-PSMA-7	65 [43–83]	77 [61–90]	56 [32–78]
AUC [95% CI]			
[^68^Ga]Ga-PSMA-11	0.63 [0.56–0.70]	0.65 [0.57–0.73]	0.65 [0.58–0.71]
[^18^F]DCFPyL	0.54 [0.45–0.62]	0.63 [0.52–0.74]	0.61 [0.53–0.69]
[^18^F]PSMA-1007	0.60 [0.46–0.73]	0.60 [0.44–0.76]	0.68 [0.55–0.82]
[^18^F]-JK-PSMA-7	0.56 [0.36–0.76]	0.57 [0.34–0.80]	0.69 [0.50–0.87]

*p* = pathology; *T* = tumour-stage; *PPV* = positive predictive value; *NPV* = negative predictive value; *AUC* = area under the receiver operator curve; *CI* = confidence interval; *nc* = non-computable

An additional analysis was performed with an alternative dichotomization regarding miT3a-stage: Likert scale points 0 (definite miT2) and 1 (possible miT3a) were treated as negative for miT3a-stage, and Likert scale point 2 (probable miT3a) as positive for miT3a stage. This analysis yielded an observer-average sensitivity, specificity, and accuracy of PSMA PET/CT of 36%, 81% and 0.58 (95% CI 0.54—0.63) for pT3a-stage and 45%, 82%, and 0.64 (95% CI 0.59—0.68) for overall ≥ pT3-stage respectively (Supplementary Table 1).

### Observer variability of molecular imaging PSMA PET T-staging

Hundred cases were re-assessed as well as dually assessed, and available for the calculation of intra- and interobserver agreement of miT-stage assessment, respectively.

For the assessment of overall T3-stage on PSMA PET/CT imaging, a substantial intra-observer agreement was found with an average Cohen’s kappa value of 0.70 (95% CI 0.60—0.81) across the four readers. Likewise, for the assessment of ≥ T3b-stage on PSMA PET/CT imaging, a substantial intra-observer agreement was found with a Cohen’s kappa value of 0.75 (95% CI 0.55—0.96).

For the assessment of overall T3-stage on PSMA PET/CT imaging, a moderate interobserver agreement was found with a Cohen’s kappa value of 0.47 (95% CI 0.33—0.61). For the assessment of ≥ T3b-stage on PSMA PET/CT imaging, a moderate interobserver agreement was found as well with a Cohen’s kappa value of 0.41 (95% CI 0.13—0.70).

## Discussion

The presence or absence of locally advanced disease (≥ T3-disease) in patients with newly diagnosed PCa is of importance for risk stratification, therapeutic decision making, and for the prediction of oncological outcome. Besides conventional clinical variables such as the determination of PSA levels, clinical tumour stage based on digital rectal examination and prostatic transrectal ultrasound, prostate MRI has been established as a pivotal and most accurate diagnostic instrument to determine local tumour stage [7]. Although MRI has uniformly shown excellent specificity for locally advanced disease, the sensitivity of MRI to predict locally advanced tumour stage (≥ T3-disease) was reported to be limited and heterogeneous among studies [4]. Therefore, there is still an unmet need to improve the prediction of final pathological tumour stage in patients newly diagnosed with PCa. Recently, PSMA PET/CT received increasing attention regarding its value in detecting the dominant cancer lesion in patients with newly diagnosed PCa and for its diagnostic performance in predicting local disease extent [20]. In the present multicentre study, reporting on 600 patients undergoing RARP for locally confined PCa and who were preoperatively staged by PSMA PET/CT imaging, the diagnostic performance of PSMA PET/CT for local tumour stage in the radical prostatectomy specimen was studied as the reference standard. The sensitivity, specificity, and diagnostic accuracy (AUC) of PSMA PET/CT were 58%, 59% and 0.59 for pT3a-stage, 30%, 97% and 0.64 for ≥ pT3b-stage, and 68%, 61% and 0.64 for overall ≥ pT3-stage disease.

PSMA PET/CT is increasingly used as a metastatic screening tool in patients with a primary diagnosis of PCa [7] and may indeed be of value for the assessment of the local extent of the primary prostate tumour as well. Woo et al. included in a systematic review and meta-analysis a total of twelve studies: ten prospective single centre studies, one prospective single centre study and one dual centre study on the PET assessment of local staging [8]. The reported pooled sensitivity and specificity of PSMA PET, using exclusively ^68^Ga-based PSMA PET tracers, were 72% (95% CI 0.56–0.84) and 87% (95% CI 0.72–0.94) for pT3a-stage disease, and 69% (95% CI 053–0.81) and 94% (95% CI 0.90–0.96) for pT3b-stage disease, respectively. The number of patients in these studies ranged from 21 to 140 and therefore was relatively small. The authors further showed that PSMA PET/MRI might have an improved diagnosed performance for local tumour stage compared to PSMA PET/CT imaging, although the number of studies using PSMA PET/MRI was only low. It is reasonable that combining different imaging modalities improves the diagnostic performance over a single imaging modality. This meta-analysis does not, however, address which imaging modality contributed preferentially to the observed improved diagnostic performance for predicting local tumour stage as compared to either of the two single imaging modalities alone. A further setback of the included studies in the meta-analysis is that often PSMA PET/CT scans were assessed by a single observer only and observer agreement scores were not provided, as opposed to the present study. This is important as interpretation of scans might differ substantially between observers. Finally, the meta-analysis by Woo et al. [8] only addressed the diagnostic performance of ^68^Ga-labelled PSMA radioligands and not that of ^18^Fluoride-labelled PSMA radioligands. This is a limitation as a large proportion of the presently performed PSMA PET scans are based on ^18^Fluoride-labelled radioligands, e.g., [^18^F]-JK-PSMA-7, [^18^F]DCFPyl, and [^18^F]PSMA-1007.

In our study, the diagnostic accuracy of PSMA PET/CT for the prediction of locally advanced disease (≥ pT3 stage) was lower than reported in smaller, initial studies and in the meta-analysis by Woo et al. [8], but was comparable to that in the recent, larger prospective study by Sonni et al. [21]. In this study comparing PSMA PET, MRI and histopathology for local tumour staging in 74 patients, the AUC for detection of extracapsular extension (ECE) was 0.59 and 0.63 for seminal vesical invasion (SVI).

Differences in diagnostic performances between studies may be due to differences in study population, differences in study set-up, differences in PSMA-radioligands and PET image acquisition, differences in the interpretation and reporting of PSMA PET/CT-based disease parameters, and in differences in the reporting of radical prostatectomy specimens. Possibly a more controlled single centre, single PSMA-radioligand, and single PET/CT system setting may have led to higher accuracy rates for predicting locally advanced disease as reported in initial PSMA PET/CT studies performed by Fendler et al. (accuracy 71% for ECE and 86% for SVI) and von Klot et al. (sensitivity and specificity 90% and 90% for ECE, respectively 75% and 100% for SVI [8, 17, 22]. It remains yet largely unknown which of the above listed variables are responsible for the reported differences in diagnostic performance between studies.

In the present study, the observers used a consensus-based standardized reporting system for molecular imaging local tumour stage, which optimized agreement of interpretation. However, relevant differences were found between intra-observer and interobserver agreement rates. The intra-observer agreement for PSMA PET/CT assessment of overall ≥ T3 stage was substantial (k 0.69), whereas the interobserver agreement for the assessment of overall ≥ T3 stage was moderate (k 0.45). Likewise, a substantial intra-observer agreement on ≥ T3b stage (k 0.75) was found, whereas the interobserver agreement for the assessment of ≥ T3b stage was moderate (k 0.41). The substantial intra-observer agreement scores suggest that further improvements in interobserver agreement may be reached by stricter adherence to image interpretation consensus criteria, which may add to the reliability of PSMA PET/CT for local tumour staging.

In comparison, Sonni et al. using a 5-point Likert scale found poor interobserver reliability rates for PSMA PET/CT assessment of ECE (intraclass correlation coefficient (ICC) 0.203) and SVI (ICC 0.081) [21]. In this study the T-staging was assessed visually in a binary manner, leaving no room for equivocal results which may have contributed to the lower interobserver agreement rates compared to our results. Muehlematter et al. reported a fair interobserver reliability for ECE (0.40; 95% CI 0.33–0.47) and SVI assessment (0.33 (95% CI 0.17–0.25) for PSMA PET/MRI, which did not differ significantly from MRI as single modality [23].

In theory, local prostate cancer staging may be hampered by the vicinity of intense urinary tracer activity in the bladder. Therefore, there may be an advantage in local prostate cancer staging for PSMA PET tracers that exhibit lower urinary excretion such as [^18^F]PSMA-1007, compared to the primarily urinary excreted tracers [^18^F]DCFPyL, [^18^F]-JK-PSMA-7 and [^68^Ga]Ga-PSMA-11. We found a lower sensitivity and diagnostic accuracy of [^18^F]DCFPyL versus [^68^Ga]Ga-PSMA-11 specifically for detecting pT3a disease. This possibly may be attributed to higher urinary tracer activity in [^18^F]DCFPyL compared to [^68^Ga]Ga-PSMA-11 [24], but other factors may play a role as well. Moreover, the difference was not statistically significant, so no definitive conclusions can be drawn from these data.

Earlier studies point to an advantage of lower urinary activity levels in local staging performance mainly in the biochemical recurrence setting (especially after prostatectomy), and not so much in the primary setting with the prostate still in situ [25–29]. In our experience, T-stage assessment in the primary setting is not hampered by urinary activity in most cases. Still, the occasional smaller tumour at the basal part of the prostate or tumours with bladder invasion may be better staged with lower urinary activity levels. Therefore, strategies to reduce urinary activity (including tracer selection and forced diuresis) in PSMA PET [24–31] may be beneficial in miT-stage assessment as well.

Strengths of the current study are the number of included patients and the comparison with histopathology as reference standard. Furthermore, patients were includedin a high-volume, dedicated multicentre prostate cancer network with a prospectively maintained database and analysed by nuclear medicine physicians with ample experience in PSMA PET reading according to E-PSMA guidelines [14]. The mix of different tracers, protocols and PET/CT systems reflects variations among clinical practices and increases generalizability of our results. Next to the strength of this analysis, it is also one of the limitations of our study. As we have used real-world data with different PSMA tracers, PET/CT systems and protocols this may have influenced the outcomes. However, PET/CT systems, protocols and image reconstructions adhered to EARL standards and EANM guidelines [10–12, 16], keeping data heterogeneity within boundaries. Other limitations include the retrospective character of this study, the observation that only a subset of 100 PSMA PET-scans were re-assessed and dually assessed which may have led to larger confidence intervals in the reported observer agreement rates, and the fact that a small proportion (3.5%) of patients were excluded because of a PSMA negative primary tumour, as it is yet unknown whether this apparent absence of PSMA expression is related to imaging technical factors or whether it is due to tumour-associated factors. Differences in the case-mix offered to the nuclear medicine physicians may have influenced the interobserver agreement rates.

Addition of PSMA PET/CT based local tumour staging might contribute to the existing nomograms for the prediction of locally advanced disease in the radical prostatectomy specimen (i.e., pT-stage disease). Further research may be aimed at improving qualitative interpretation criteria as well as possible addition of quantitative parameters which may improve diagnostic accuracy and interobserver agreement rates.

## Conclusion

In a large, multicentre study evaluating 600 patients with newly diagnosed with intermediate and high-risk prostate cancer, we showed that PSMA PET/CT may have a valuable role in local tumour staging when pathological tumour stage in the radical prostatectomy specimen was used as the reference standard. The interobserver and intra-observer variability of tumour extent on PSMA PET/CT was moderate to substantial.

### Supplementary Information

Below is the link to the electronic supplementary material.

Supplementary file1 (DOCX 4209 KB)

## Data Availability

The datasets generated and analysed during the current study are not publicly available as these contain individual person’s data but are available from the corresponding author on reasonable request, after pseudonymization of the data and legal agreement.
