# Supplementary material for: The accuracy and intra- and interobserver variability of PSMA PET/CT for the local staging of primary prostate cancer
Source: Eur J Nucl Med Mol Imaging. 2024 Jan 26;51(6):1741–52. doi: 10.1007/s00259-024-06594-0 (PMC11043118; doi:10.1007/s00259-024-06594-0)
Supplement: Supplementary file 1 — Supplementary file1 (DOCX 4209 KB) [file 259_2024_6594_MOESM1_ESM.docx]

Supplementary Table 1.

Additional analysis with alternative dichotomization: Likert scale points 0 and 1 were treated as negative, and 2 as positive for miT-stage 3a. Observer-average diagnostic accuracy of PSMA PET/CT based tumour stage (miT-stage) for pathological tumour stage (pT-stage) in 579 patients with a PSMA-positive prostate undergoing pre-operative PSMA PET/CT imaging and concurrent robot assisted radical prostatectomy (RARP) as reference standard, regarding **A**. pT3-stage and **B**. ≥pT3-stage.

| **A.** | | | | |
| --- | --- | --- | --- | --- |
|  |  | **no pT3a; *n*** | **pT3a; *n*** | **Total; (PPV/NPV)** |
| no miT3a | | 292 | 139 | 431 (68% NPV) |
| **miT3a** | | 69 | 79 | 148 (53% PPV) |
| Total | | 361 (81% specificity) | 218 (36% sensitivity) | 579 |
|  | | AUC 0.58 [95% CI 0.54 - 0.63] | |  |
|  | |  |  |  |
| **B.** | | | | |
|  | | **pT2; *n*** | **≥pT3; *n*** | **Total; (PPV/NPV)** |
| miT2 | | 183 | 195 | 358 (48% NPV) |
| **≥miT3** | | 40 | 161 | 201 (80% PPV) |
| Total | | 223 (82% specificity) | 356 (45% sensitivity) | 579 |
|  | | AUC 0.64 [95% CI 0.59 - 0.68] | |  |
| p = pathology; mi = molecular imaging; T = tumour-stage; PPV = positive predictive value; NPV = negative predictive value; AUC = area under the receiver operator curve | | | | |
